# Generative AI and large language models in health care: pathways to implementation

**DOI:** 10.1038/s41746-023-00988-4

**Published:** 2024-03-07

**Authors:** Marium M. Raza, Kaushik P. Venkatesh, Joseph C. Kvedar

**Affiliations:** Harvard Medical School, Boston, MA USA

**Keywords:** Health policy, Health care economics

## Abstract

Generative AI is designed to create new content from trained parameters. Learning from large amounts of data, many of these models aim to simulate human conversation. Generative AI is being applied to many different sectors. Within healthcare there has been innovation specifically towards generative AI models trained on electronic medical record data. A recent review characterizes these models, their strengths, and weaknesses. Inspired by that work, we present our evaluation checklist for generative AI models applied to electronic medical records.

## Introduction

In November 2022, OpenAI launched ChatGPT, an artificial intelligence (AI) chatbot and search tool. ChatGPT is a tool that uses generative AI: AI that is designed to create or generate new content, such as text, images, or music from trained parameters^1^. Tools like ChatGPT use “large language models” (LLMs), multi-layer neural networks that are trained on large amounts of data to simulate human conversation^2^. Other LLM tools include Google’s Bard, Microsoft Bing, Chatsonic, Github Copilot, and ChatSonic to name just a few. LLMs themselves are an example of a “foundation model,” a broader term for an AI model trained on a large quantity of data at scale.

The buzz around generative AI has skyrocketed, with ChatGPT expanding to have over 100 million users^3^. Over the past year, many have shown excitement for potential generative AI applications to healthcare. LLMs have already been used to pass the United States Medical Licensing Examination^4^, write research articles^5^, and interpret electronic medical record data^6^. This last use case is perhaps closest to the bedside. Generative AI models trained on EMR data, such as notes, lab values, and billing codes, hold the promise of better predictive performance, simpler model development (with less labeled data required) and cheaper model deployment^7^. At the same time, those critiquing the utility of these applications have argued that generative AI is simply another health-tech fad, with many roadblocks preventing its implementation^8^. One valid concern for example is regarding generative AI models ‘hallucinating,’ or inventing responses when they do not have sufficient information^9^.

## Evaluating generative AI models for EMRs

In their review, Wornow et al. explore the current state of generative AI models for EMRs^7^. Specifically, Wornow et al. conducted a review of 84 foundation models trained on clinical structured text data from EMRs. This is the largest review of foundation models within health care to date. To define the key characteristics of the models, Wornow et al. make the distinction between (i) clinical language models, which intake, and output clinical text, and (ii) EMR models that intake a patient’s entire EMR to output a machine-understandable ‘representation’ for a patient, similar to a digital twin^10^.

Wornow et al. found evidence that both types of models enable more accurate model predictions, but authors also found limitations. Currently, nearly all clinical text models are trained on either a single relatively small database or the entirety of PubMed. The ‘representation’ models are trained on small public datasets only or a single private healthcare system’s EMR. Thus, Wornow et al. found that current uses of generative AI within healthcare are limited by their lack of generalizability and issues of data privacy. Specifically, AI models based on data from different EMR systems are not generalizable, and very few AI models have had details, such as model weights, published due to data privacy concerns. Additionally, minimal work has been conducted to validate whether other, potentially more valuable benefits of FMs will be realized in healthcare (Table 1).

**Table 1:** Generative AI model evaluation checklist

Predictive performance & Multimodality	Auto-draft response accuracy (i.e. Epic system) measured Predicted orders/labs accuracy (i.e. Oracle Cerner system) measured Accuracy rate stratified by data type i.e. text vs voice vs image vs video Manual user correction rate measured Model hallucination rate quantified	☐ ☐ ☐ ☐ ☐
Less labeled data	Cost/volume of training data required before clinical use calculated Implementation time (from model acquisition to clinical use) measured	☐ ☐
Simplified model deployment	Cost of model implementation calculated Clinician hours/administration hours saved calculated Training time and training resources before clinical use quantified Cost of technological support needed during clinical use calculated	☐ ☐ ☐ ☐
Emergent clinical applications	Number of new/innovative clinical applications identified	☐
Novel human-AI interface	Clinician satisfaction surveyed Patient satisfaction surveyed Qualitative points of feedback synthesized into report for model improvement	☐ ☐ ☐

Noting those limitations, Wornow et al. propose an improved framework to evaluate generative AI models for healthcare settings. They elaborate upon six criteria: predictive performance, data labeling, model deployment, emergent clinical applications, multimodality, and novel human-AI interfaces. By evaluating models around these criteria, Wornow et al. argue that health systems will be better able to judge which are best for more stratified clinical needs.

## Applying the Wornow et al. framework

This work comes at a time when exciting new EMR LLMs are being launched. For example, in April Microsoft announced a partnership integrating its OpenAI service with the Epic EHR. This collaboration may involve using generative AI to auto-draft responses to common and/or time-intensive patient messages^11^. Oracle Cerner has also integrated generative AI into its EHR. Recently, (Nov 2023), Oracle Cerner announced the Oracle Clinical Digital Assistant tool, a multimodal voice and screen-based tool that will participate in appointments by automating notetaking and proposing actions such as medication orders, labs, and follow-up appointments. Providers should be able to talk to the tool to access elements of a patient’s EHR, while patients should be able to talk to the tool to book appointments and ask questions^12^. In deciding to implement either Epic or Oracle Cerner’s generative AI applications, Wornow et al.’s evaluation framework becomes important to assess each model’s true clinical value. Below is a checklist to be used while conducting a model evaluation, stemming from Wornow et al.’s six points. This type of checklist could be modified based on a specific generative AI model or clinical setting, and then could be used regularly for model evaluation.

## The pathway forward: leadership, incentives, and regulation

The improved evaluation framework Wornow et al. propose is one important step forward. To truly make generative AI more than just a fad within healthcare, a broader pathway to implementation is required. This pathway must include defined leadership for development, adoption incentives, and continued regulation.

Leadership will be required first and foremost to push continued model development, validation, and implementation. Currently, generative AI models have been developed by startup companies, research groups, as well as academic healthcare systems. Given these varied developers, guidance from a leadership body is needed to clarify the practical path towards implementation. Leadership should focus on developing guidelines for model performance (i.e. minimizing model hallucination), data sharing, finding the optimal healthcare settings for clinical trials using generative AI tools, as well as clarifying the needs of different healthcare settings (i.e. community vs. academic, private vs. public institutions). Ideally, this type of leadership will come from an organization involving physicians, healthcare administrators, developers, and investors. A sub-committee within the FDA could be well positioned to undertake such responsibility.ßß

Alongside leadership, continued regulation will be required to balance the interests of developers, healthcare systems, payers, and patients. The continued evaluation of tools based on frameworks such as that developed by Wornow et al. must be conducted on the scale of individual health institutions so that tools with clinical relevance are prioritized. On the larger scale, as with other AI tools, policies surrounding liability, data privacy, and bias within predictive modeling must be clarified before insights from generative AI tools can be put into practice. While the FDA has begun to adapt its regulatory framework to address AI technology as medical devices it must move from discussion to action, and provide specific guidance for LLMs^13,14^. The FDA can learn from the strengths as well as the criticisms of the EU’s AI Act, one of the first formal regulations for generative AI^15^.

Finally, as with any other healthcare technology, payer incentives must be present before widespread implementation. Generative AI tools will likely be considered a capital expense in the books of most providers and can follow along the same or similar financing path as EHR systems. Additionally, given that the cost to create and evaluate generative AI tools remains somewhat prohibitive, both private and public investment will be required to truly push the field forward.

The time is now to capitalize on the excitement around generative AI and LLMs. The weakness of the generative AI space that Wornow et al. highlight, including those around model generalizability and evaluation, should be taken as guideposts for improvement. With leadership, incentivization, and regulation, generative AI within healthcare can be put on a feasible pathway for implementation.

## Data Availability

No datasets were produced or analyzed for this article.

## Code availability

No computer code was produced or analyzed for this article.
